# Stable Isotope Analysis Provides New Information on Winter Habitat Use of Declining Avian Migrants That Is Relevant to Their Conservation

**DOI:** 10.1371/journal.pone.0034542

**Published:** 2012-04-05

**Authors:** Karl L. Evans, Jason Newton, John W. Mallord, Shai Markman

**Affiliations:** 1 Department of Animal and Plant Sciences, University of Sheffield, Sheffield, United Kingdom; 2 UK NERC Life Sciences Mass Spectrometry Facility, SUERC, East Kilbride, United Kingdom; 3 Royal Society for the Protection of Birds, The Lodge, Sandy, Beds, United Kingdom; 4 Department of Biology, University of Haifa – Oranim, Tivon, Israel; Ben-Gurion University of the Negev, Israel

## Abstract

Winter habitat use and the magnitude of migratory connectivity are important parameters when assessing drivers of the marked declines in avian migrants. Such information is unavailable for most species. We use a stable isotope approach to assess these factors for three declining African-Eurasian migrants whose winter ecology is poorly known: wood warbler *Phylloscopus sibilatrix*, house martin *Delichon urbicum* and common swift *Apus apus*. Spatially segregated breeding wood warbler populations (sampled across a 800 km transect), house martins and common swifts (sampled across a 3,500 km transect) exhibited statistically identical intra-specific carbon and nitrogen isotope ratios in winter grown feathers. Such patterns are compatible with a high degree of migratory connectivity, but could arise if species use isotopically similar resources at different locations. Wood warbler carbon isotope ratios are more depleted than typical for African-Eurasian migrants and are compatible with use of moist lowland forest. The very limited variance in these ratios indicates specialisation on isotopically restricted resources, which may drive the similarity in wood warbler populations' stable isotope ratios and increase susceptibility to environmental change within its wintering grounds. House martins were previously considered to primarily use moist montane forest during the winter, but this seems unlikely given the enriched nature of their carbon isotope ratios. House martins use a narrower isotopic range of resources than the common swift, indicative of increased specialisation or a relatively limited wintering range; both factors could increase house martins' vulnerability to environmental change. The marked variance in isotope ratios within each common swift population contributes to the lack of population specific signatures and indicates that the species is less vulnerable to environmental change in sub-Saharan Africa than our other focal species. Our findings demonstrate how stable isotope research can contribute to understanding avian migrants' winter ecology and conservation status.

## Introduction

A new global phenomenon appears to be emerging in the extinction crisis, that migratory species are declining much more rapidly than residents [1]. Some of the clearest evidence for this concerns long-distance avian migrants. Species that breed in Europe and winter in sub-Saharan Africa are declining to a significantly greater extent than residents and short-distance migrants that use similar resources during the breeding season [2]. The mechanisms driving these declines are poorly understood. Whilst changes in the quality of breeding grounds can be important, there is increasing evidence that many migrant populations are regulated by wintering conditions through direct influences on survival or indirect impacts on reproductive success through carry over effects [3]–[9].

Knowledge of African wintering conditions is important for understanding migrant population dynamics for many species, but despite this even basic information about winter ecology, such as habitat selection, is lacking. For almost all species the extent of migratory connectivity between geographically isolated breeding populations is poorly understood [7], [9]. Habitat selection data are essential when assessing if habitat loss and degradation contribute to migrant population declines. Migratory connectivity assessments are useful for addressing a wide range of ecological and evolutionary questions, and for assessing if populations that share wintering grounds exhibit similar population trajectories, which is the predicted pattern if winter conditions are the primary determinant of population trends [10]. Many factors contribute to the lack of data on habitat selection and migratory connectivity, but amongst the most important are the very small proportion of birds ringed on the breeding grounds that are recovered in sub-Saharan Africa, major spatial biases across sub-Saharan Africa in the probability of such ringing recoveries, and insufficient field observations of the least well known species [7], [11].

The wood warbler *Phylloscopus sibilatrix*, house martin *Delichon urbicum*, and common swift *Apus apus* are three declining African-Eurasian migrants whose wintering ecology is particularly poorly known, although data are also insufficient for many other species. Wood warbler ringing recoveries from sub-Saharan Africa are almost entirely lacking, and thus the wintering locations of specific breeding populations are unknown [11], [12]. The species is infrequently sighted on the wintering grounds, and whilst it is one of the few migrants observed in lowland moist forest [13], [14] the species' winter habitat requirements are generally considered very poorly understood [15]. The species is of conservation concern across Europe due to rapid declines across much of their distribution, for example British populations have declined by 63% between 1995 and 2009 [16], [17].

House martins are rarely sighted on their sub-Saharan African wintering grounds and ringing recoveries are very rare, with less than one recovery per 300,000 individuals ringed on the breeding grounds [18]. Consequently, the house martin's wintering grounds and preferred habitat type are poorly understood, although they are suspected to primarily forage over moist montane forest [13], [14], [18]. House martins have declined in a number of European countries, such as France where populations have declined by 40% during the last decade, and consequently the species is of conservation concern across Europe [16].

Common swift ringing recoveries from sub-Saharan Africa are more frequent than those of house martins or wood warblers, but they are still insufficient for assessing the wintering locations of particular European breeding populations [19], [20]. The species is considered to use a wide range of both grassland and woodland habitat types in its wintering range, but it is unknown if particular habitats are preferentially selected [14], [21]. A number of European countries have recently detected declines in common swift populations that are of sufficient magnitude to warrant designation as a species of national conservation concern, for example British populations declined by 31% between 1995 and 2009, but the species is not yet considered to be of European conservation concern [16], [17].

The analysis of stable isotope data requires careful interpretation, but the technique has regularly been successfully used to compare populations' migratory pathways and to infer habitat selection [22]. Carbon stable isotopes are especially useful for distinguishing broad habitat types in sub-Saharan Africa that differ in their proportion of C4 and C3 plants as these groups have divergent photosynthetic pathways which differentially partition stable carbon isotopes [23]. This is the dominant control of the carbon isotope composition of consumers; C3 plants generally have values of around −35‰ to −20‰, whereas C4 plants have a d^13^C range of about −18‰ to −7‰. Consequently, consumers dependent on resources originating from C3 dominated wooded vegetation tend to be depleted in ^13^C (i.e. have more negative d^13^C values) than equivalent consumers that use C4 dominated grassland habitats [23], [24]. On a smaller scale, since d^13^C is a key indicator of water-use efficiency [25], the same plants sampled in drier habitats may have d^13^C values 2 to 4‰ heavier compared with wetter areas [26]. Nitrogen isotope ratios can also indicate aridity levels (higher values indicating greater aridity [27]) but this is further complicated by the increase in d^15^N with trophic level and the influence of very local variation in anthropogenic activities such as fertiliser applications [24], [28]. Variance in stable isotope composition can be used as an indicator of isotopic niche space, thus providing some information regarding the focal population's position along the specialist-generalist continuum [29], [30]. Such interpretations must take into account the factors that can drive variation in isotopic signatures and the potential for divergent habitats to exhibit similar signatures. Therefore, across the very broad geographic distributions that are the focus of this study, interpretations of isotopic variance should be restricted to discussion of specialisation along axes relating to the use of C3 versus C4, and mesic versus xeric habitats. The comparison of stable isotope signatures from geographically isolated breeding populations has been frequently used to assess the extent to which populations exhibit migratory connectivity on the wintering grounds [10], [22]. Geographically segregated breeding populations of avian migrants that exhibit migratory connectivity, i.e. share common wintering grounds, are predicted to exhibit similar isotopic signatures in winter grown tissues. Such patterns in isotopic signatures are frequently interpreted as evidence for migratory connectivity, but they could also arise if populations winter at different locations but exploit isotopically similar diets [31]–[36].

Here, we present data on the stable isotope signatures of wood warbler, house martin and common swift feathers that were grown on the African wintering grounds and obtained from multiple breeding populations. We use these data to inform discussion of winter habitat selection and migratory connectivity of these declining species in order to inform future assessments of the potential contribution of factors operating on their wintering grounds to these declines.

## Methods

### Ethics statement

Feather sampling protocols were approved by national organisations responsible for licensing bird ringing activities and feather sampling (BTO, the Home Office, Swiss Federal Office for the Environment, Vogelschutzwarte, Israel Nature and National Parks Protection Authority). The following permits gave permission to take feather samples (UK: 40/3214, 1511, 4956, 1660; Germany: 3600SOK720-13/2009ra; Switzerland: FOEN F044-0799; Israel: 32887 32874 31871 32200).

### Feather collection

The wood warbler and house martin undergo a complete moult on the wintering grounds, whilst common swifts moult their body feathers during winter [37]–[39]. Feather samples were taken from breeding adults of each focal species in north-west and central Europe, and from Israeli breeding populations of swifts and house martins (Table 1). All samples were taken in 2009, except those from German swifts which were sampled in 2008. There are few data on how plant-based isoscapes for Africa can change through time and demonstrating the consistency of patterns over several years spanning climatic variability is thus advisable. However, high repeatability of carbon and nitrogen isotope ratios in winter grown feathers from individuals sampled in different years, or within populations sampled in different years, have been documented for many, albeit not all, African-Eurasian migrants [34], [36], [40]. Therefore, whilst further research that assesses the isotopic signature of samples across multiple years may be useful, we believe that our conclusions are not unduly influenced by collecting samples from a single year.

**Table 1:** Feather sampling site locations for breeding wood warblers, house martins and common swifts.

Sampling location	wood warbler	house martin	common swift
UK - Derbyshire	53°22′N; 01°36′W	53°13′N; 01°18′W	53°13′N; 01°18′W
- Dorset	-	50°47′N; 01°58′W	-
- mid Wales	53°06′N; 03°54′W	-	-
- Somerset	51°11′N; 03°35′W	-	-
Germany	50°30′N; 11°38′E	-	50°11′N; 8°30′E
Switzerland	-	47°11′N; 08°2′E	-
Israel	-	31°49′N; 34°51′E	31°24′N; 34°44′E

Two to three rump or upper-tail coverts were taken from each individual upon arrival on the breeding grounds, and before any post-breeding moult commenced. In addition, a one cm section of a middle secondary feather was clipped from some wood warblers. Rump and upper-tail coverts were also taken from a small number of free flying juvenile Israeli house martins to contrast with samples from adults. This was done as house martin populations breeding at relatively low latitudes can exhibit different moult strategies to those from more temperate regions and we wished to confirm that adult Israeli house martins did not moult their body feathers on the breeding grounds. All wood warblers and most house martins were sexed, except those in Israel, using behavioural and morphological criteria [37]. Swifts cannot be sexed on equivalent criteria [39].

### Stable isotope analysis

Feathers were washed in a 2∶1 chloroform ∶ methanol solution to remove surface contaminants and then air dried. Each analysis included a single clean feather. For all species, the sample included the vane and parts of the rachis to which the vane was attached. When analysing body feathers samples almost invariably included the entire feather, for wood warbler wing feathers the outermost section of the feather was consistently used. Samples were cut into small fragments and placed into a clean 3×5 mm tin capsule. Sample weights were 0.7 mg for swift feathers and 0.4 mg for the smaller wood warbler and house martin. Capsules were then combusted in a Costech ECS 4010 elemental analyser interfaced with a Thermo Delta XP Plus stable isotope ratio mass spectrometer. All stable isotope ratios are reported in permil (‰) using the δ notation:
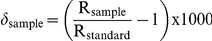
where d_sample_ is the isotope ratio of the sample relative to a standard, R_sample_ and R_standard_ are the fractions of heavy to light isotopes (e.g. ^13^C/^12^C, ^15^N/^14^N) in the sample and standard respectively. d^13^C and d^15^N are reported relative to their respective international standards, i.e. V-PDB and AIR. Repeat analysis of internal gelatin standards gave a standard deviation for d^13^C and d^15^N measurements of 0.17‰ and 0.10‰ respectively (n = 129). d^13^C was calibrated against the glutamic acid reference materials USGS40 and USGS41 [41]; d^15^N was calibrated against the glutamic acid reference materials as well as the ammonium sulphate standards IAEA-N1 and IAEA-N2 and USGS25.

### Statistical analysis

All analyses were conducted in SPSS. We first compared isotope ratios between conspecific populations. Kolmogorov-Smirnov tests showed that each variable (populations tested separately) was normally distributed with the exception of carbon isotope ratios in Israeli swifts. The frequency distribution of these data was not markedly skewed, and we thus conducted all tests assuming normal distributions. Paired t-tests were used to compare isotope ratios in the body and wing feathers collected from the same wood warbler. One-way ANOVAs were used in all other tests of differences in isotope ratios between populations followed by a post-hoc multiple comparison test. We used the Scheffe post-hoc test when populations exhibited equality of variance (assessed using Levene's test), and Tamahane's T2 post-hoc test when populations exhibited inequality of variance. To assess the extent to which species share isotopically similar resources we compared inter-specific differences in the isotopic breadth of resources used with Levene's test for differences in the variation in isotope ratios, and then assessed inter-specific differences in stable isotope ratios using ANOVA. Means are consistently reported ± one standard error.

## Results

### Wood warbler

Wing and body feathers collected from the same individual exhibited very similar carbon (mean difference 0.41‰±0.31; paired T-test: T = 1.35, P = 0.19, n = 29) and nitrogen isotope ratios (mean difference 0.15‰±0.14; paired T-test: T = 1.04, P = 0.31, n = 29). All subsequent analyses were thus conducted using isotope data from body feathers as these were available from more individuals.

**Figure 1:**
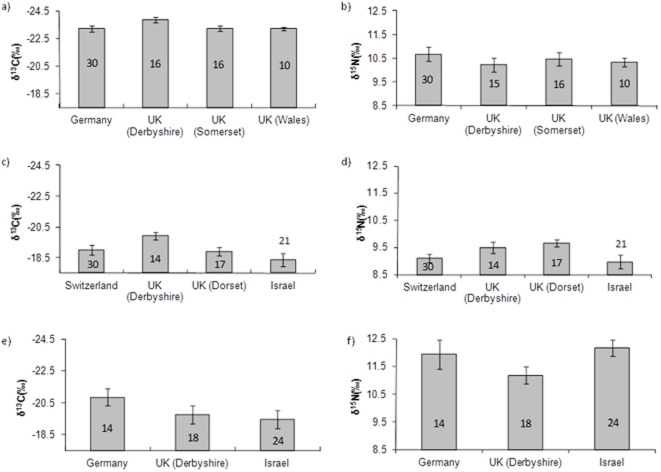
Carbon and nitrogen isotope compositions in winter grown feathers of wood warblers (a, b), house martins (c, d) and common swifts (e, f) sampled from different regions of the breeding distribution Error bars represent one standard error, and numbers represent sample size.

Different populations exhibited similar variances in carbon isotope ratios (F_3,68_ = 2.24, P = 0.09), but significant differences in the variance of nitrogen isotope ratios (F_3,68_ = 3.47, P = 0.02) with the Welsh population exhibiting the lowest variance (3.41‰). Carbon isotope values did not exhibit significant differences between samples from different regions (F_3,68_ = 1.73, P = 0.17, n = 72; Fig. 1a), or genders (F_1,69_ = 0.03, P = 0.86, but only 12 females were sampled). Nitrogen isotope values did not differ significantly between breeding populations (F_3,68_ = 0.49, P = 0.69, n = 72; Fig. 1b) or genders (F_1,69_ = 0.39, P = 0.54). The mean carbon and nitrogen isotope ratios across all populations were respectively −23.41‰±0.10 and 10.48‰±0.16 (n = 72).

### House martin

Body feathers from juvenile (n = 5) and adult (n = 21) house martins sampled on the Israeli breeding grounds differed significantly in their carbon (juvenile mean: −22.80‰±0.10; adult mean −18.38‰±0.41; F_1,24_ = 26.74; P<0.0001) and nitrogen isotope ratios (juvenile mean: 10.79‰±0.22; adult mean 8.97‰±0.25; F_1,24_ = 11.52; P<0.002). Whilst isotopic compositions of juvenile feathers may in part be influenced by growth rates and physiological processes linked to development, these results support the hypothesis that adult Israeli house martins moult their body feathers away from the breeding grounds, as do populations breeding at higher latitudes. Therefore, all adult samples from the Israeli population were included in subsequent analyses.

All sampled populations exhibited similar variances in their carbon isotope ratios (F_3,78_ = 2.18, P = 0.10). The influence of region on carbon isotope ratios (Fig. 1c) was not significant, although α was close to the standard threshold of significance (F_3,78_ = 2.65, P = 0.06, n = 82); Scheffe's post-hoc test did not demonstrate significant differences between any populations (P values range from 0.12 to 0.96). We interpret these data as evidence that there was not any biologically significant variation in carbon isotope ratios between our sampled populations. When we excluded the Israeli samples and eight samples from Dorset (UK) that were not sexed, the effect of gender was not significant (F_1,49_ = 2.87, P = 0.10). Populations exhibited similar variances in their nitrogen isotope ratios (F_3,78_ = 2.67, P = 0.06). Region had a marginally significant influence on nitrogen isotope ratios (F_3,78_ = 2.79, P = 0.05), and when excluding samples that were unsexed, gender was not significant (F_1,49_ = 3.56, P = 0.07). Scheffe's post-hoc test showed no significant differences between most populations (P values range from 0.35 to 0.99) but found a marginally significant difference (P = 0.05) between populations breeding in northern England (mean 9.48±0.21, n = 14) and Israel (mean 8.97±0.25, n = 21). The differences between these means are greater than the measurement error (0.17‰) but they are small (0.51, Fig. 1d) and only marginally significant. We thus interpret these data as evidence that there was not any biologically significant variation in nitrogen isotope ratios between our sampled populations. Across all populations the mean carbon isotope ratio was −18.99‰±0.18 and the mean nitrogen isotope ratio was 9.25‰±0.20 (n = 82).

### Common swift

Levene's tests indicated that populations exhibited similar variances in their carbon (F_2,53_ = 1.36, P = 0.27) and nitrogen (F_2,53_ = 1.64, P = 0.20) isotope ratios. Swifts cannot be reliably sexed on morphological characters and thus the influence of gender cannot be assessed, but there were no significant differences between sampled populations' carbon (F_2,53_ = 1.40, P = 0.25, n = 56) or nitrogen isotope ratios (F_2,53_ = 2.15, P = 0.13, n = 56; Fig. 1e,f). The mean carbon and nitrogen isotope ratios were respectively −19.89‰±0.34 and 11.78‰±0.21 (n = 56).

### Cross species comparisons

**Figure 2:**
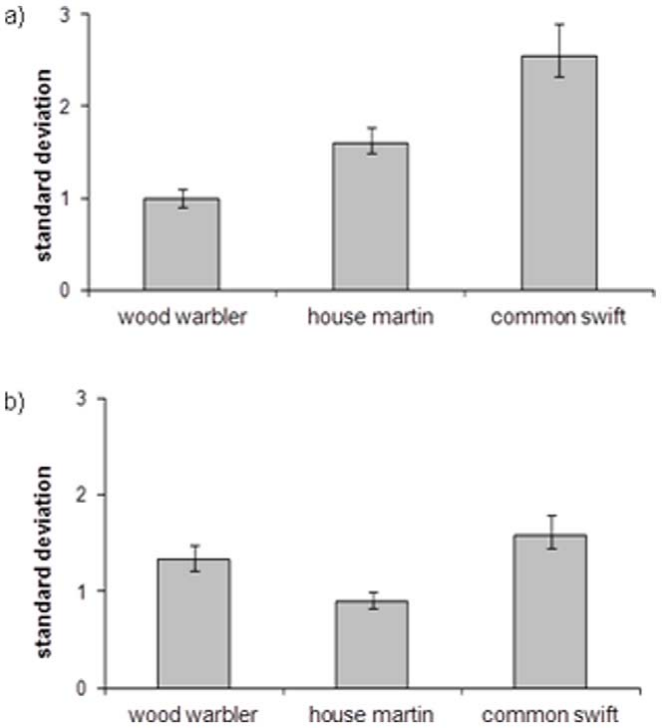
Standard deviation in (a) carbon and (b) nitrogen isotope ratios in winter grown feathers sampled from breeding wood warblers (n = 72 and 71 for carbon and nitrogen respectively), house martins (n = 82) and common swifts (n = 56). Data are pooled across conspecific breeding populations as these do not exhibit significant differences in their variances;error bars represent one standard error.

Variance in carbon isotopes exhibited significant differences between species (F_2,206_ = 36.65, P<0.0001), with wood warblers exhibiting significantly lower variance than house martins (F_1,151_ = 11.67, P = 0.001), which in turn exhibited significantly lower variance than swifts (F_1,136_ = 26.97, P<0.0001; Fig. 2a). Variance in nitrogen isotopes exhibited significant differences between species (F_2,206_ = 14.51, P<0.0001); house martins exhibited significantly lower variance than wood warblers (F_1,151_ = 8.89, P = 0.003), which in turn exhibited significantly lower variance than swifts (F_1,125_ = 5.28, P = 0.023; Fig. 2b).

**Figure 3:**
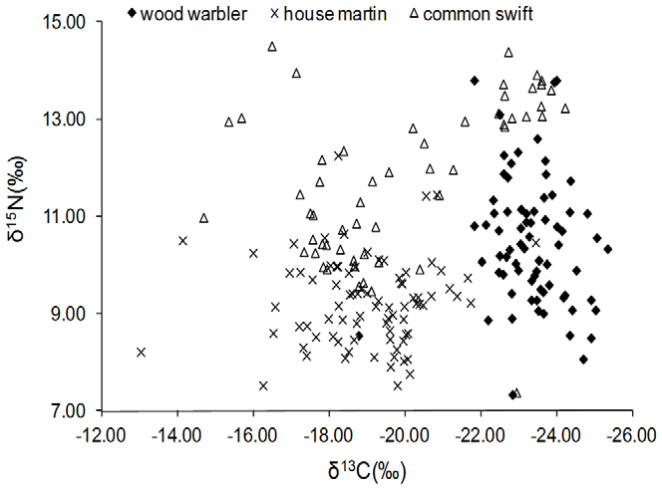
Carbon and nitrogen stable isotope profiles of winter grown feathers of wood warbler (n = 71), house martin (n = 82) and common swift (n = 56).

Mean carbon isotope ratios differed between species (F_2,206_ = 124.85, P<0.0001) with Tamhane's T2 multiple comparison test revealing significant differences between wood warblers and house martins (P<0.0001), wood warblers and common swifts (P<0.0001), and a marginally non-significant difference between house martins and common swifts (P = 0.06; Fig. 3). Mean nitrogen isotope ratios differed between species (F_2,206_ = 68.08, P<0.0001) with Tamhane's T2 multiple comparison test revealing significant differences between all species pairs (P<0.0001; Fig. 3).

## Discussion

Conspecific wood warbler, house martin, and common swift populations breeding in distinct regions of the western Palearctic did not exhibit significant differences in the carbon or nitrogen isotope ratios of feathers grown on the wintering grounds. Divergence in stable isotope ratios is typically interpreted as evidence for a lack of migratory connectivity, although it is possible that divergence could be generated if populations shared wintering regions but differed in habitat use or diet [31]–[36]. The similarity in isotope ratios that we detect could thus arise from geographically isolated conspecific breeding populations sharing common wintering locations, i.e. strong migratory connectivity, or similarity in habitat use/diet across spatially isolated locations.

Wood warbler wing and body feathers exhibited statistically identical stable isotope ratios, suggesting that habitat use is similar during the six week period in which these feather tracts are typically moulted [37]. Wood warblers exhibited limited variance in their carbon and nitrogen isotope ratios, particularly in comparison with the high levels of intra-specific variance in equivalent signatures of aquatic warbler feathers grown at a single site in west Africa [42]. This limited variance could arise because during the winter moult wood warblers use a restricted range of habitats along the continuum of C3 to C4 and mesic to xeric vegetation types, or because they occupy a wider range of habitats but specialise on food items that are derived from specific vegetation types along these axes of niche space. Both of these mechanisms indicate that wood warblers are specialists. This specialisation probably contributes to the lack of divergence in isotope ratios of populations breeding in different European regions and our data are thus insufficient to assess the magnitude of migratory connectivity in our sampled wood warbler populations. Wood warblers' carbon isotope ratios (mean −23.4‰) are amongst the most ^13^C-depleted reported for African-Eurasian migrants wintering at similar latitudes, many of which are considered to primarily use relatively dry savannah woodland incorporating a mix of C3 trees and C4 grassland (e.g. willow warbler *Phylloscopus trochilus*: population means −21.7‰ to −12.9‰ [31], [34]; wryneck *Jynx torquilla*: −20.9‰ to −18.3‰ [36]; sand martin *Riparia riparia*: c. −20.0‰ [43]; collared flycatcher *Ficedula albicollis*: c. −19.0‰ [35]; reed warbler *Acrocephalus scirpaceus*: −19.1‰ [44]; savi's warbler *Locustella luscinioides*: −16.4‰ [44]; barn swallow *Hirundo rustica*: −14.8‰ [32]. Indeed, wood warbler carbon isotopic signatures are similar to those of American redstart *Setophaga ruticilla* (−24.5 to −24.0‰), an insectivorous species that winters in wet lowland tropical forest [45]. This similarity in signatures is increased when adjusting for variation in carbon fractionation factors between tissue types [46] (2.7‰ for feathers; 1.7‰ for the blood used in the American redstart study).The more ^13^C-depleted wood warbler signatures seem likely to arise from use of resources obtained from habitat types that are dominated by C3 vegetation [23], [24] and that are relatively wet [25], [26], providing further support for previous suggestions that wood warblers may rely on moist woodland [13], [14]. Indeed, recent sightings on the wintering grounds have been largely confined to the wettest patches in the transition zone between closed evergreen forest and more open savannah woodland (J Vickery & C Orsman pers. comm.). Regardless of the precise nature of wood warblers' sub-Saharan habitat type, we provide strong evidence that wood warblers specialise on a limited range of resources during the moulting period, and their populations are highly likely to be sensitive to environmental change on their wintering grounds which reduces the availability of these resources.

It has previously been suggested that in sub-Saharan Africa, the house martin primarily forages over the canopy of moist montane forests [13], [14], [18]. We find that house martins' carbon isotope ratios (mean −19.0‰) are similar to the carbon isotope ratios documented in a number of African-Eurasian migrants (see above) that primarily use relatively dry open habitats [7]. House martins' carbon isotope ratios would be expected to be more depleted if they primarily used moist woodlands, which are dominated by C3 vegetation. It thus seems, in contrast to previous suggestions, highly improbable that house martins rely on moist forests and changes in the availability or quality of such habitats are unlikely to be major drivers of house martin population declines. Nitrogen stable isotopes are frequently used to assess trophic levels; house martins have lower d^15^N values than those of swifts, which is compatible with house martins feeding at lower trophic levels, but numerous other factors including variation in fertiliser use and aridity could drive this pattern [24], [27], [28]. House martins' stable isotope signatures exhibit significantly less variance than those of common swifts, despite both species being aerial insectivores and being sampled at geographically similar breeding locations. These data thus suggest that during the winter house martins either use a more restricted range of habitat types or, given the marked spatial variation within sub-Saharan Africa in the relative proportion of C3 and C4 plants [47]–[49] that house martins occupy a smaller geographic range than the common swift. As habitat specialists and species with smaller geographic ranges are more vulnerable to environmental change [50]–[52], we suggest that house martin populations are more vulnerable to changing conditions on the African wintering grounds than the common swift.

Common swift populations exhibit the highest variance in carbon and nitrogen stable isotope ratios, the variance in the former is particularly notable (−14.7‰ to −24.2‰). Such a pattern could potentially arise if the species winters in a small range of habitat types that contain both C3 and C4 dominated vegetation, but that some individuals predominantly feed on insects exploiting C3 vegetation (generating ^13^C-poor signatures) whilst others primarily feed on insects exploiting C4 vegetation (generating ^13^C-rich signatures). This seems improbable as aerial insectivores, including the common swift, are opportunistic feeders that are highly unlikely to select insects according to the type of vegetation that the insects, or the insects' prey, feed on [53]–[54]. The high variance in carbon isotope ratios could also be generated if swifts primarily foraged on emerging aquatic insects, as there can be remarkable variation in carbon isotope ratios of lake ecosystems [55]. This seems unlikely to be the primary cause of the large variation in swifts' carbon isotope ratios as whilst aquatic systems, within the swift's wintering range, receive much attention from birdwatchers only a relatively small proportion of observations of wintering swifts relate to such habitat types [14], [21]. It seems much more plausible that the marked variance in swifts' carbon and nitrogen stable isotope ratios arises because the species forages over a wide range of vegetation types that differ in their proportions of C3 and C4 plants. Such variation could arise because swifts exploit a wide range of habitat types within a relatively small wintering range, or because swifts use a more restricted range of habitat types but these vary markedly in the dominance of C3 and C4 plants, for example grasslands in southern sub-Saharan Africa are increasingly dominated by C4 vegetation [47]–[49]. Both the use of a wide range of habitats and a large geographic range during the winter will reduce the susceptibility of common swift populations to environmental change in sub-Saharan Africa compared to more specialised species.

Here, we demonstrate how stable isotope signatures can provide important data regarding the winter ecology of declining long distance migrants that, in combination with other information, facilitates assessment of the threats migrants face during the non-breeding period. Despite much progress in the development of light-weight tracking devices, such as geo-locators, these are still expensive to deploy on a large number of individuals, are currently too heavy for use on many of the smaller passerines, and the spatial accuracy of fixes (c. 200 km) [56] prevents accurate description of habitat selection in the numerous regions in which habitat types vary over finer spatial scales. Therefore, despite challenges of using stable isotopes to track migratory species [22], [42] they can still contribute to understanding the winter ecology and conservation requirements of a wide range of migratory bird species.
